# Artificial Intelligence in Physical Sciences: Symbolic Regression Trends and Perspectives

**DOI:** 10.1007/s11831-023-09922-z

**Published:** 2023-04-19

**Authors:** Dimitrios Angelis, Filippos Sofos, Theodoros E. Karakasidis

**Affiliations:** Condensed Matter Physics Laboratory, Department of Physics, University of Thessaly, Lamia, 35100 Greece

## Abstract

Symbolic regression (SR) is a machine learning-based regression method based on genetic programming principles that integrates techniques and processes from heterogeneous scientific fields and is capable of providing analytical equations purely from data. This remarkable characteristic diminishes the need to incorporate prior knowledge about the investigated system. SR can spot profound and elucidate ambiguous relations that can be generalizable, applicable, explainable and span over most scientific, technological, economical, and social principles. In this review, current state of the art is documented, technical and physical characteristics of SR are presented, the available programming techniques are investigated, fields of application are explored, and future perspectives are discussed.

**Supplementary Information:**

The online version contains supplementary material available at 10.1007/s11831-023-09922-z.

## Introduction

Data science has been the driving force for the dawn of the fourth industrial revolution, bringing the big-data concept in focus of most science and engineering applications. It has been stated that the global datasphere will get as high as 175 Zettabytes by the year 2025 while it was merely 33 Zettabytes in 2018 [1]. As a result, researchers from most disciplines have been motivated on exploring ways to deploy this vast amount of data. The main idea is the identification of patterns and/or hidden equations that govern these datasets. Data mining, apart from the exploitation of well-established statistical and numerical methods, has also been directed towards the extraction of mathematical expressions, which can be utilized for the establishment of new and the verification of existing physical laws. Therefore, the question that now arises is: Should we acknowledge the era we experience as the era of big data, and if so, what is the effect on science and technology?

**Fig. 1:**
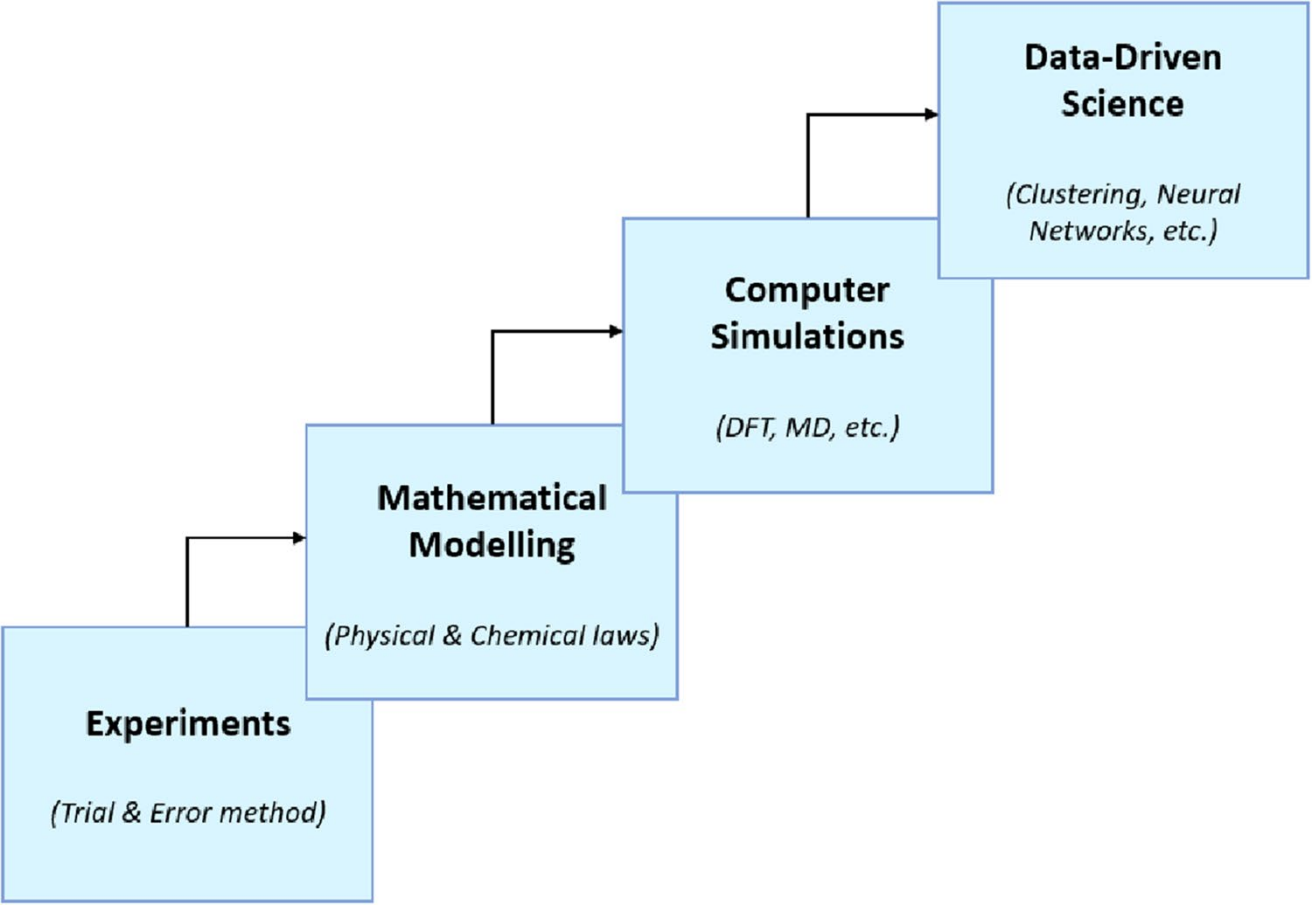
The four paradigms of science: empirical, theoretical, computational and data-driven

Novel data-driven approaches are now exploited in materials science, among others, [2] (see Fig. 1) and have opened the road to the introduction of Materials Informatics [3, 4] and relevant approaches whose primary concern is the discovery of novel materials at reasonable computational cost. Furthermore, in order to enhance this initiative, there exist databases (e.g., Inorganic Crystal Structure Database [5], Open Quantum Material Database [6], The Cambridge Structural Database [7], AFLOWLIB [8]) to provide adequate support for scientists and engineers. However, materials science isn’t the only field that has benefited from the advent of big data. The fourth paradigm of science, under the framework of Artificial Intelligence (AI) to facilitate the procedure [9], has substantially evolved in fields such as bioinformatics, particle physics, space research, medical imaging, construction applications, and more. To support this observation, the universal recognition of big data and data-driven methods is further becoming clear by the vast increase of published articles on the topic [10].

Nevertheless, big data would be hard to handle without the incorporation of AI, or, in other words, the assemblage of methods and skills that allow humans and machines to execute assignments that only intelligent entities can (e.g., perceive, reason, and act) [11]. Having been introduced as a branch of computer science, AI is further comprised of techniques with their own categories of algorithms (for example, Machine Learning and Particle Swarm Optimization [12]), suggesting a statistical, predictive framework that can be bound to a specific problem both in research and in everyday life.

Machine Learning (ML) is an AI technique that has acquired major interest for data analysis tasks, based on its ability to learn from experience and, therefore, provides accurate approximations and/or predictions on the underlying patterns [13]. Oftentimes, common statistical analysis has been misidentified to ML, however, the former aims on the accurate description of observations while the latter is constructing algorithms whose focus lies on successful data classification and approximations in order to predict the outcome [14]. ML is widely recognized as an effective tool for research and applied purposes, in fields like additive manufacturing [15, 16, 17, 18], materials science [14, 19, 20, 21, 22, 23, 24, 25, 26, 27, 28, 29], autonomous driving [30, 31, 32], solar cells [33, 34, 35, 36, 37, 38, 39], chemistry [40, 41, 42], welding industry [43], solar radiation [44, 45, 46, 47, 48, 49, 50, 51] and many more.

Notwithstanding the data-driven techniques evolution, barriers have arisen when it comes to the interpretation of the underlying physics, that has somehow to be extracted from data patterns. A successful attempt to bind physical laws to the solution of partial differential equations has been made with the incorporation of Physics-Informed Neural Networks (PINNs) [52] and some of their new implementations, such as PI-GP [53] and B-PINNs [54]. From another point of view, during the development of a NN the output triggered by an input value is predetermined, and that means the mapping could be abstract [55]. Although this is a procedure that machines can deal with, it might be extremely difficult for humans to make a sense out of it [56], since this “black-box” model [57] does not adhere to the principles of explainable and generalizable AI. Several problems may also come up when these models are used in the field, for instance, when a manufacturer of an autonomous car cannot apprehend the choice that the car will do in infrequent critical situations (as in protecting the driver or pedestrians in an imminent crash), concerns might appear on the applicability of the algorithm [58]. The absence of a physical interpretation in data-driven black-box models, has been the driving force for a change of heart.

**Fig. 2:**
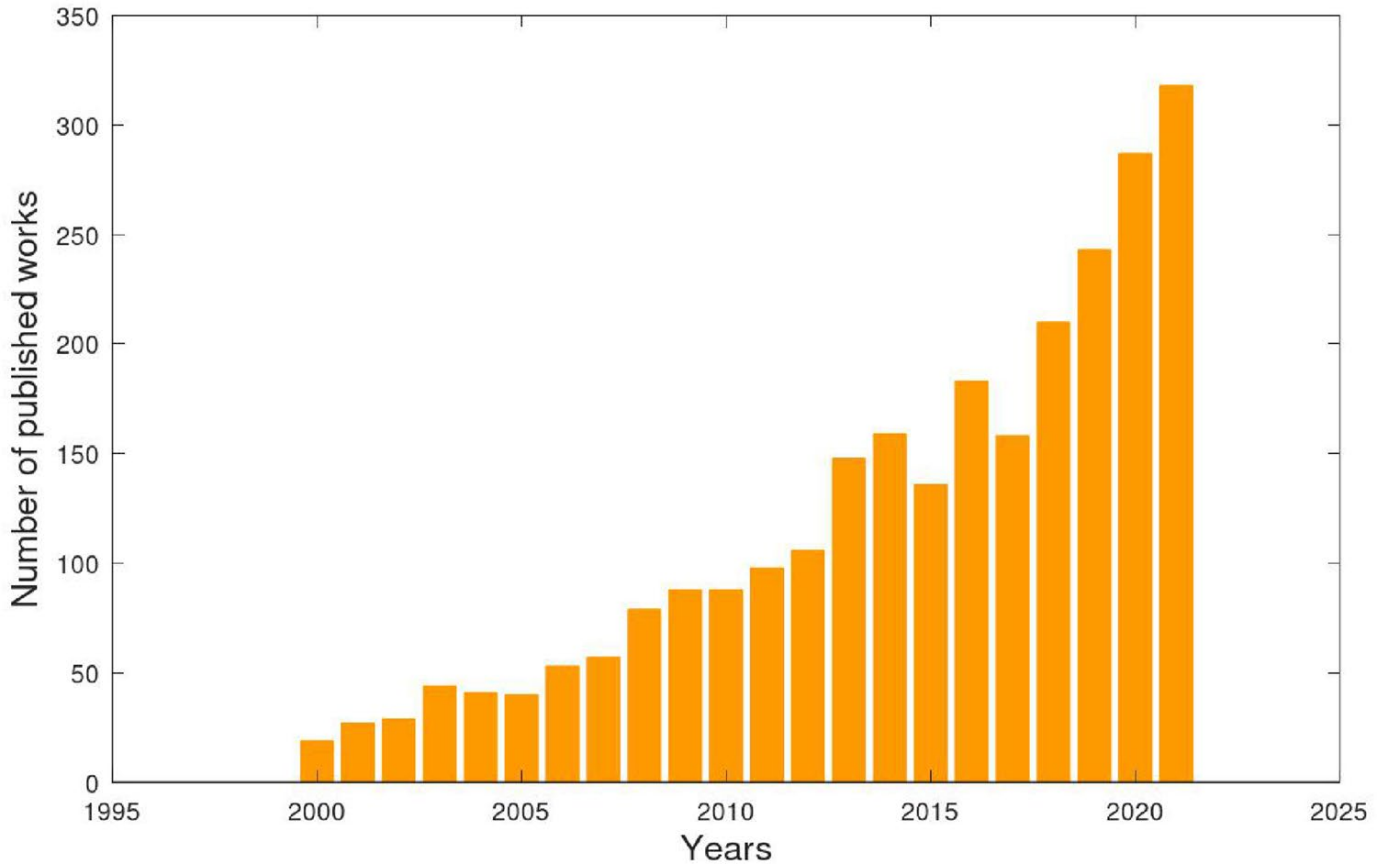
Publications related to symbolic regression from 2000 to 2021

This review has been focused on presenting an ML-based method, Symbolic Regression (SR), which has been developed on Evolutionary Computing principles. SR is differentiated from a purely data-driven black-box model, as it is equipped with the ability to generate symbolic expressions (analytical equations) without considering prior constraints and provides a physics-inspired overlook. The ever growing adaptation of SR in various scientific fields has been captured by the rapid growth of related published papers in the last decade and more (see Fig. 2) and it would be beneficial to dive deep into its remarkable characteristics that have made it a new trend in physics-based computations. In the sections that follow, there will be a brief introduction on various ML types (Sect. 2) adopted in science and engineering, a comprehensive analysis of SR and the respective algorithms incorporated (Sect. 3), mapping of scientific fields where SR has been successfully employed and others that have shown a promising prospect (Sect. 4) and, finally, we sum up with a concluding discussion and present the future perspectives of SR (Sect. 5).

## Machine Learning

The present section is organized as follows. Firstly, categories and useful information about ML will be introduced. Secondly, there will be a brief presentation of several ML algorithms that researchers are most familiar with. Finally, more complex approximations such as Deep Learning will be discussed.

### Machine Learning Categories

Machine Learning can be subdivided into three major categories, (i) Supervised Learning (SL), (ii) Unsupervised Learning (UL) and (iii) Reinforcement Learning (RL) [59], although the latter isn’t always recognized as a separate division. In SL, the training procedure involves data manipulation based on labeled input/output pairs, while the implied algorithms seek for determining a hidden function able to map input behavior to the desired output. On the other hand, in UL, no labels are specified on the input data and the algorithmic procedures urge to reveal the implied data interconnections [60]. In RL, the model does not require input data as it constructs its own by self-training and, furthermore, it is self-challenged to achieve higher accuracy metrics [61]. A combination of SL and UL has also been proposed in Semi-Supervised Learning (SSL) in which both labeled and unlabeled data are utilized. SSL focuses on the identification of how the learning procedure may be affected by a mixture of labeled and unlabeled data and the construction of algorithms capable of exploiting this scheme [62].

A successful ML algorithm implementation is tightly paired with the quality and quantity of available data. In cases where data extracted from various databases and/or literature sources poses no viable option, attention is drawn into experimental or simulation output (e.g., Density-Functional Theory (DFT), Molecular Dynamics (MD), etc.) [23], posing a four-way route to acquire data. Attention has to be drawn also on data representation, as it may vary from discrete (e.g., texts) to continuous (e.g., vectors and tensors) or weighted graphs [22]. Still, data availability is not enough; it is imperative to follow a common format [63] and, most of the times, a pre-processing step is required [23].

However, an inherent disadvantage of such approaches is bound to the fact that ML methods are prone to overfitting. Overfitting occurs when an algorithm is “finely” trained on a specific dataset, which in turn results on high statistical errors when applied to a different dataset. In such case, the proposed algorithm becomes unsuitable for further use, or in other words, ungeneralizable. To overcome this issue, various techniques have been proposed, such as hold-out, k-fold cross-validation, and regularization [16].

### Machine Learning Algorithms

There has been a wealth of ML algorithms being established throughout the years, from simple linear models to laborious deep learning architectures. Some of the most widely adopted are Artificial Neural Networks (ANN), Support Vector Machines (SVM) and Decision Trees (DT) based. The idea behind ANN is that it reacts the same way as the neural networks of human brain, with its abilities spanning to applications such as classification, regression, learning and generalization [64]. SVM models are utilized for classification, while its regression counterpart is the Support Vector Regression (SVR) model [28]. DT methods employ tree-form graphs and have been utilized for classification tasks. DTs are susceptible to complexity and overfitting issues, and various alternatives have been constructed, such as Random Forest (RF) and Gradient Boost (GB), by employing different trees in a forest or a serial weighted manner, respectively [14]. There are many references in the literature for these models, which are not going to be covered here (see, for example, [65]).

Nevertheless, although implementations based on Shallow Learning algorithms, such as ANNs, SVMs, and DTs, are quite effective in numerous fields (e.g., materials science), some issues may occur in demanding applications, such as poor accuracy over DFT simulations [22]. This has opened the discussion on establishing more robust methods, such as Deep Learning (DL).

### Deep Learning

While Shallow Learning methods may be effective and accurate on dealing with data over a small set of computational nodes, DL is capable of exploiting big data by mapping it to multiple layers in order to extract information and make predictions [23], even on noisy data [66]. DL models embed mathematical concepts (linear algebra, probability theory) and programming techniques in hidden layers that span over a number of thousands or more [12, 21], making them perfectly fit in applications ranging from processing videos and images [67, 68, 69, 70], speech recognition [71], and bio-informatics [72], among others. Nevertheless, physical interpretation of the outcome still lacks, and this would benefit their application in physical sciences, where interpretability has a central role. In such cases, it would be beneficial to adopt models that produce meaningful results (i.e., mathematical expressions), which could spot correlations with existing empirical relations and propose an analytical approach bound to physical laws.

## Symbolic Regression

Symbolic Regression is a type of regression analysis in which a mathematical function that describes a given dataset is derived. While conventional regression methods (e.g., linear, quadratic, etc.) have their independent variable(s) predetermined and try to adjust a number of numerical coefficients in order to achieve perfect fit, SR attempts to find the parameters and equations simultaneously [55].

Derived from the superset of AI available methods, SR is usually implemented by evolutionary algorithms. At the same time, the most widely adopted concepts for SR construction are adopted from Genetic Programming (GP) [73]. In this section, the basic features of GP will be presented, an analytical development of the basic SR procedure is to be exemplified, several features that characterize SR superiority will be presented, while available SR programming techniques will be evaluated.

### Genetic Programming Fundamentals

**Fig. 3:**
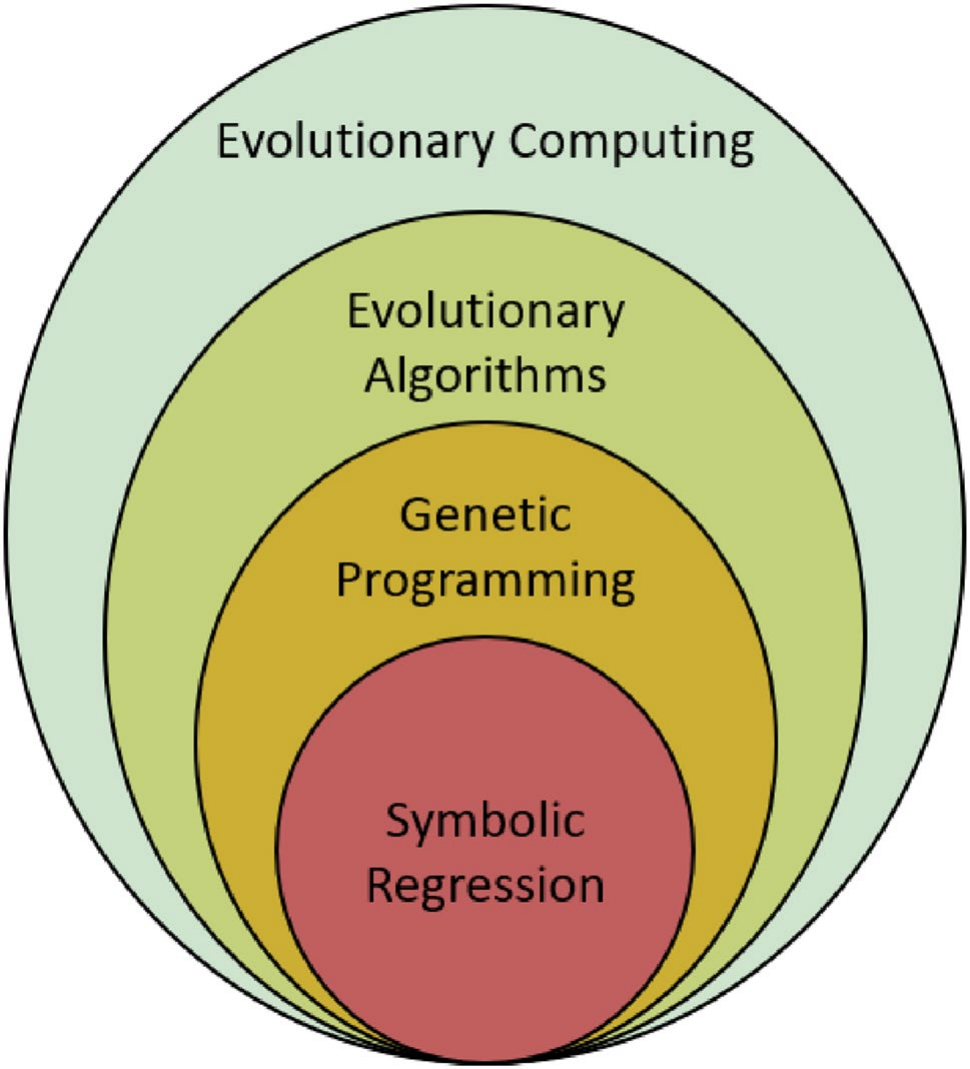
From evolutionary computing to symbolic regression

Genetic Programming (GP) [74] is an Evolutionary Algorithm (EA) instance [75] which in turn is a subset of Evolutionary Computing (EC) [76] (see Fig. 3). Moreover, GP provides the framework to express data behavior through mathematical equations, by exploring the available mathematical space in an evolutionary process. It is a fact that GP can find applicability in most regression-based science and engineering problems. The procedure that GP follows, includes the construction of different symbolic expressions, on which a comparison is made on its parts. The expressions that do not comply with accuracy and complexity measures being set are discarded, while those that appear as a potential solution to the problem are combined and form an output expression able to produce the desired outcome. The most common way to visualize a symbolic expression is a tree-structure with nodes and branches. Currently there are numerous programming options that can implement GP calculations, such as the Glyph package in Python [77] and GPTIPS toolbox in MatLab [78].

### GP-SR Procedure

**Fig. 4:**
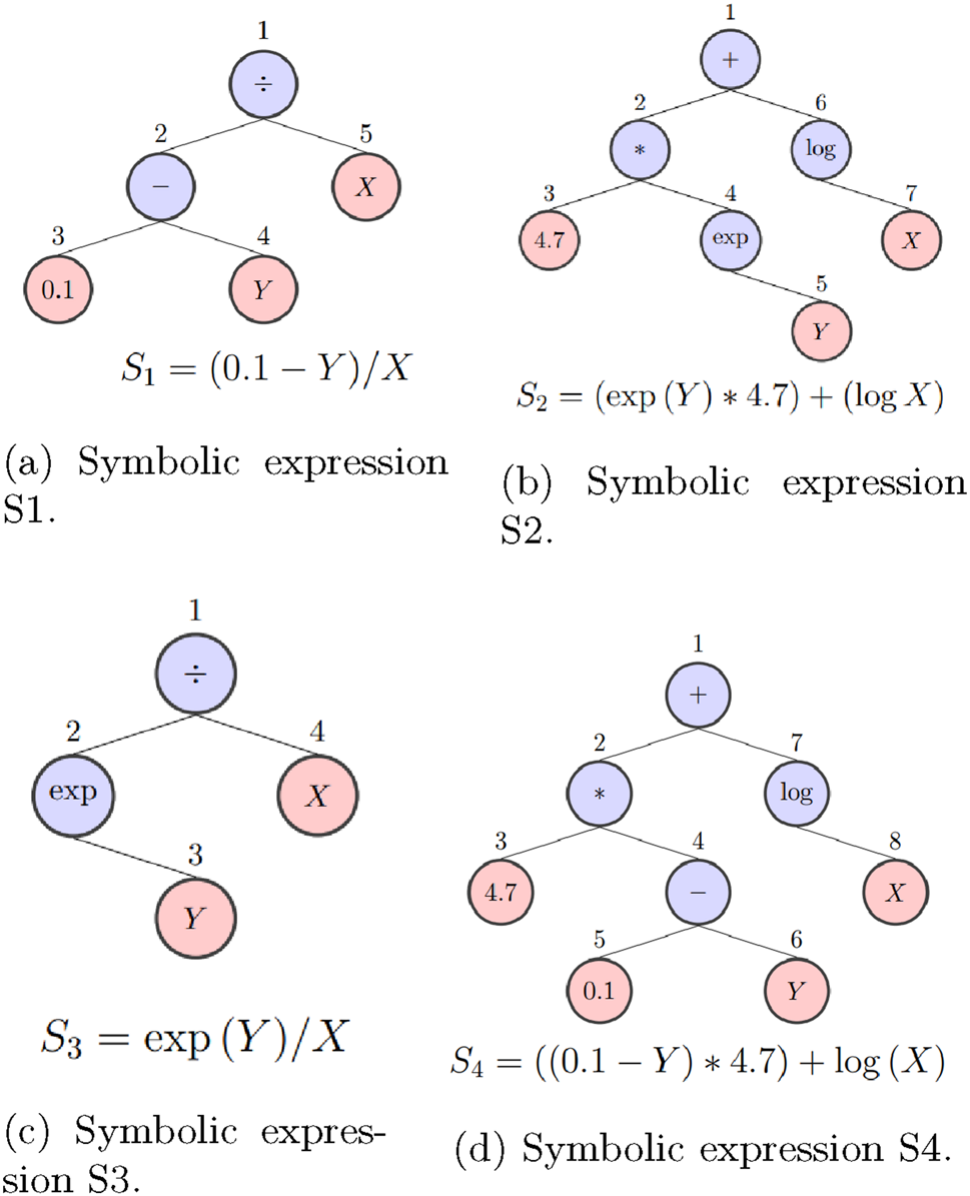
Symbolic expression examples

The GP tree-structure scheme consists of primitive functions and terminal nodes. While primitive functions could appear in any possible form (e.g., +, −, $*, \div , \text {log}$, etc.), terminal nodes correspond to data inputs (e.g., *X,Y*) and numeric constants (e.g., 0.1) that may be needed to construct a symbolic expression. The final combination gives the desired expression in the form of a rooted tree. For example, in Fig. 4a, a tree-structure that corresponds to the symbolic expression $S_{1}$ is presented. The numbers that appear above each node are irrelevant to the procedure and serve only as a reference point. Nodes with number 1 and 2 are primitive functions (e.g., $\div$, −) while nodes 3,4,5 are filled with a numerical constant (e.g., 0.1) and input variables (e.g., *X, Y*). Configurations of primitive functions and terminals are drawn hierarchically in GP, while at the same time they serve as “individuals” from a population that contains a plurality of those.

The process taking place aims on finding the proper number of nodes/terminals that achieve the best fit over a given dataset and correspond to a final equation. For that reason, GP evolves by exploring every possible implementation between the primitive functions and terminals. To gain insight on the complexity implied, it should be noted that the search takes place in an infinite space that includes all available mathematical operators and numerical constants. Therefore, to search for the optimal fit, comparison measures are required, and these are related to the nature of the problem investigated. During an equation search, each possible implementation is rated by the number of data it can effectively handle, and the one that seems more likely to contribute on the final equation is evaluated on the error it produces. A common error metric usually incorporated in SR algorithms is the sum of the squares (*SSE*), derived from the differences between the predicted outcome and the tabulated output value [56], given by:

1 $$\begin{aligned} SSE=\sum _{i=1}^{n} (Y_{i}-\hat{Y_{i}})^{2}~, \end{aligned}$$

or due to the fact that the search occurs on various instances, the error could be measured as an average (Mean Squared Error (*MSE*)), with a mathematical formula of:

2 $$\begin{aligned} MSE=\frac{1}{n} \sum _{i=1}^{n} (Y_{i}-\hat{Y_{i}})^{2}~, \end{aligned}$$

where, *n* is the total number of inspected situations, i corresponds to the individual situation, $\hat{Y}$ is the predicted value of the configuration and *Y* is the correct answer.

Starting from the top, from an infinite po*o*l of choices, GP creates the first population randomly, while the size of the population is predetermined by the user. Each configuration inside the population could possibly contribute as a poor fitness parameter, affecting the whole dataset. However, some configurations might appear more effective than others. By continuously examining the effect of each one of these implementations, the process discovers promising candidates that produce small error, and employs them in future implementations, while those with poor performance are abandoned. Furthermore, the selection of those parts (sub-configurations) are random (e.g., sub-configuration 2-3-4 from Fig. 4a), resulting that way on a generated symbolic expression that differs in terms of tree shape and depth compared to the parental expression.

For instance, let there be another symbolic expression $S_{2}$ as shown in Fig. 4b. Additionally, let $S_{2}$ be deemed efficient by the comparison on fitness and therefore sustain the combination as described above. Finally, let the node with number 2 from Fig. 4a (sub-configuration 2-3-4) and the node with number 4 from Fig. 4b (sub-configuration 4-5) be randomly selected for the combination. Then, the configurations in the symbolic expression $S_{1}$ and $S_{2}$ have their sub-configurations exchanged. When the exchanging procedure is completed, two new configurations have emerged by the parts of their parents, while the parental configurations are concurrently removed from the procedure. Thus, new symbolic expressions are obtained, $S_3$ and $S_{4}$, which are depicted in Fig. 4c and d respectively. Although their shape has changed and contributed to the creation of new expressions, the fact that their depth remained the same is purely coincidental, as the investigation of possible configurations and combination of those, occurs on thousands of expressions. It should also be noted that the exchange takes place in parallel and by an iterative manner, random parts of promising expressions are combined till the final goal of obtaining a robust equation.

The evolutionary procedure proceeds step by step, as the average error is decreased due to the removal of poor-performing expressions. Eventually, at a predetermined point, the GP sequence finalizes, and the equation is exported. Reasons for termination could be either the definition of a maximum step on the algorithm (i.e., a limit set on the created populations by the user), a point where the statistical error of an individual equals to zero (optimal equation) or a point where the statistical error is lower than a given constant (e.g., 0.01). However, a mixture of those is preferred to cover every aspect. Finally, it should be noted that it is not mandatory for GP to produce one equation as it can be defined to export a number of suggestions, usually with a ranging complexity between equations. Here, it has to be beard in mind that low complexity might indicate poor error performance, while high complexity value could be prone to overfitting (Pareto front) [56].

**Fig. 5:**
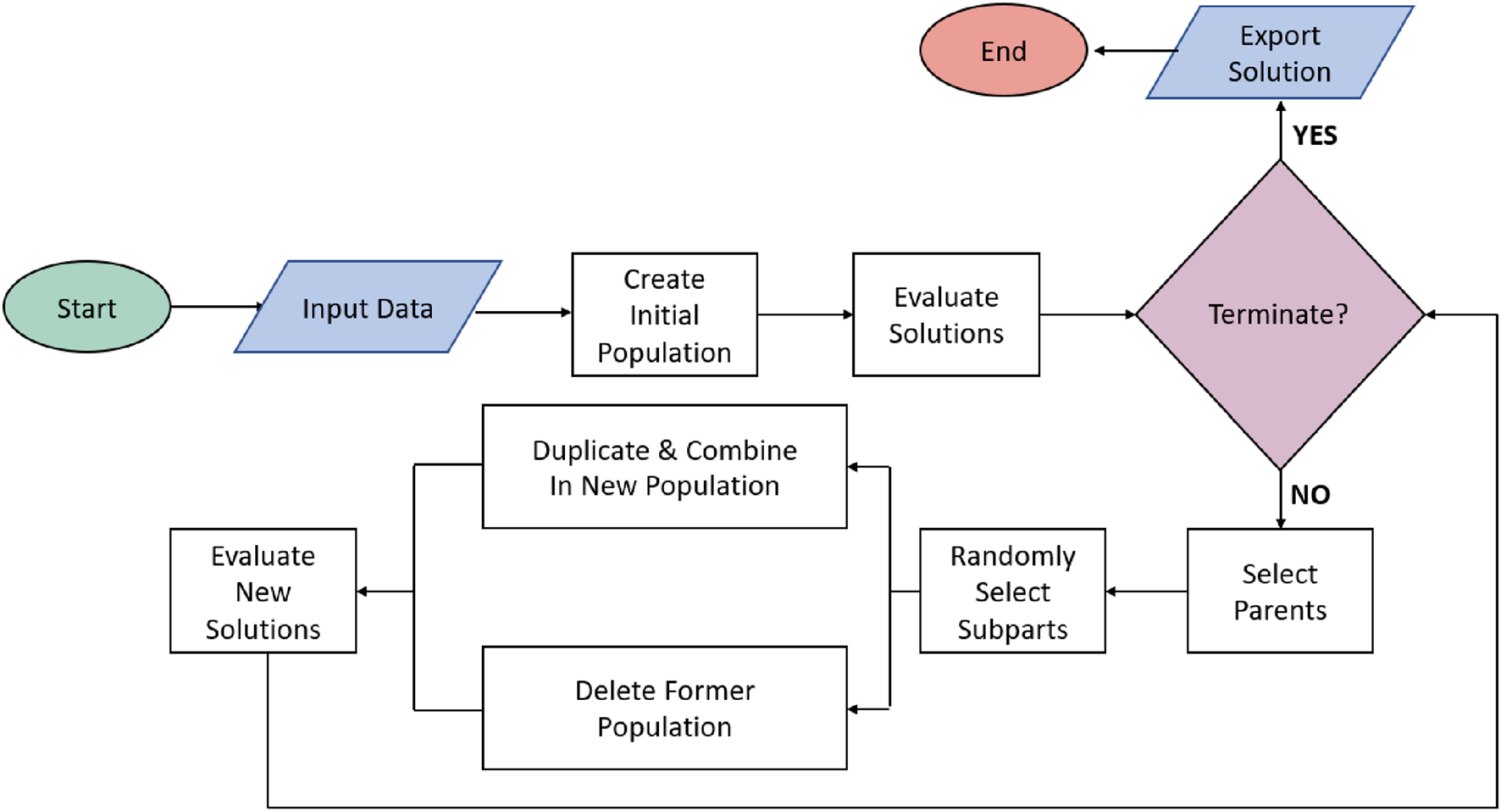
Genetic programming based symbolic regression flowchart

In other words, GP initiates the procedure by creating an initial population filled by random symbolic expressions, with dimensions that vary according to the user configuration. Random mutations take place to minimize the possibility of the algorithm being trapped in local minima [79]. A way to visualize symbolic expressions is by a tree-structure form that contains primitive functions and terminal constants. Additionally, by producing a great number of expressions, GP compares them on the basis of how thy fitting on the given dataset. Candidate expressions that achieve small error are selected for further investigation. It should be reminisced that the nature of the comparison varies according to the problem’s domain, while in the present case, the *SSE*, or the *MSE* are employed. Furthermore, GP surveys all possible sub-configurations and combines them in a way that creates a combined superset, whereas the parental expressions are discarded, and the final equation(s) are exported. A flowchart that employs GP evolution is illustrated in Fig. 5.

### Programming Techniques

During the past decades, the extreme computational cost of SR implementations has been the main reason that posed barriers on its wide adaptation. Nowadays, as hardware has evolved in fast parallel architectures, the road for new SR approaches has opened, suggesting both efficiency and calculation speed.

At present, there are ample methods in the literature that propose different SR implementations. To mention a few, there exist models that employ a Monte Carlo tree search (MCTS) algorithm [80], a matrix-based encoding process [55], and advanced pre-processing schemes applied before algorithmic training [81] or even before regression begins [82]. Others have generated algorithms such as nearest neighbor indexing [83] or non-evolutionary techniques such as the FFX algorithm [73]. The latter, although extremely fast, produces non-interpretable equations [84]. In addition, there also exist Mixed-Integer Non-Linear Programming (MINLP) formulations [85, 86], models that identify the SR problem as a linguistic [87], others that incorporate probabilistic features such as probabilistic framework [88] or probabilistic grammars [89], Bayesian approaches [90] and more [91, 92].

The concept of increased accuracy is the focal point of GP approaches as it constitutes a key element on the applicability of ML models [93]. There have been efforts on modifying the basic GP-SR procedure [18, 94, 95, 96, 97, 98], while, on the other hand, some argue that GP-based procedures lead to abstract mathematical formulas that make no sense [83]. An intriguing idea that is also supported is the restriction of the search space into a set of symbols, by incorporating several constraints into the algorithm (usually by accepting prior knowledge about the system) [55, 58, 89, 99, 100]. Towards this direction, one could enforce a certain pattern to the generated expressions, such as monotonicity or symmetry and, as a result, increase the accuracy, reduce the computational cost, while, in parallel, adhere to well-established physical empirical or analytical expressions that already exist in the literature and need modifications according to the problem under investigation [101].

However, researchers should be aware of the limitations implied in such approaches. For instance, a non-GP approach [84] which constraints the derived formula to a set of mathematical symbols, has produced accurate predictions with low computational cost, but, on the downside, the algorithm has shown limited applicability and adaptability, since it cannot employ more complex cases, where periodicity [84] or exponentiality is needed. Hybrid methods have also been proposed, such as Deterministic/GP-SR models [102], neural-guided/GP [103] and others [99, 104, 105, 106]. Moreover, Neural Network based architectures have been employed [107, 108], while others have gone a step further and merged NN-based models with several DL features [109, 110].

Promising results have been obtained through methods that incorporate Bayesian Neural Networks (BNN) [111]. Bayesian statistics [112] in contrast to conventional statistics (also known as frequentist statistics) do not consider a fixed parameter, but they rather identify it as a random variable which can be described with a probability distribution. BNN functions as a typical Neural Network with the exception that the parameters are distributions, instead of a fixed value, and the training occurs via Bayesian inference. This is an important feature of BNN as it provides the ability to quantify uncertainties, meaning that the algorithm incorporates confidence intervals instead of a single point. Moreover, Bayesian inference considers every plausible scenario that could happen, and it marginalizes the parameters over the most possible outcome. For example, in image processing, if an image appears distorted, frequentist statistical inference has no power to rationalize and make a valid sense out of it as it has no available space to work. It is what it is. On the other hand, Bayesian inference, model the problem by navigating through probabilities. Thus, it overcomes the issue generated by the distorted (noisy) image [112].

At present, there are various programming techniques to implement SR under GP principles, either heuristic or effective only in the implied data region, being presented either as stand-alone codes or in user-friendly platforms (e.g., Eureqa [113], HeuristicLab [114]), free or commercial. A relevant list is presented in Table 1. Finally, future studies on SR can exploit the open-source characteristics of SRBench [115] initiative, which is a promising benchmark that can provide access in different datasets, perform algorithmic comparisons and result analysis, among others.

**Table 1:** List of various SR approaches

Short description	Year	References
Shape-constrained	2022	[100]
PS-Tree (GP modification)	2022	[97]
DoME (deterministic)	2022	[84]
Bayesian	2022	[90]
Tensorial sparse	2022	[92]
Mixed-integer non-linear programming	2022	[86]
Probabilistic framework	2022	[88]
Functional-Hybrid model	2022	[104]
TaylorGP (GP modification)	2022	[96]
GSR (matrix based encoding scheme)	2022	[55]
Symbolic Physics Learner (MCTS)	2021	[80]
Physically constrained	2021	[99]
GP-GOMEA (GP modification)	2021	[116]
Temporal regression	2021	[91]
SymbolicGPT (probabilistic language)	2021	[87]
Large scale pre-training	2021	[81]
Pre-regression	2021	[82]
Hybrid Neural-Guided/GP	2021	[103]
Probabilistic grammars	2021	[89]
Neural Network based	2021	[109]
GP modification	2021	[94]
Bayesian Neural Network	2021	[111]
AI Feynman 2.0 (graph modularity)	2020	[108]
GP modification	2020	[18]
Multi-task SISSO	2019	[117]
AI Feynman (Neural Network)	2019	[107]
DSR (Deep Learning)	2019	[110]
Positional CGP (GP modification)	2018	[95]
IT (search space constrain)	2018	[58]
SISSO	2018	[118]
Mixed-integer non-linear programming	2017	[85]
Hybrid	2013	[102]
FFX (fast & deterministic)	2011	[73]
Nearest Neighbor Indexing	2010	[83]
Eureqa (software)	2009^a^	[113]
HeuristicLab (software)	2002^a^	[114]

^a^First launched

### Pros and Cons

In contrast to other non-linear regression methods, SR does not require a priori knowledge of the studied system, as it is completely data-driven [119]. Of outmost importance is the fact that SR can identify ambiguous relations in datasets and therefore provide a more profound solution [80]. There may be cases that governing equations that describe a system are partially known [120], and this is also a field where SR can apply, providing a deeper understanding. Towards generalizable AI, it provides a closed-form mathematical expression easier to incorporate at models similar to the one under investigation (e.g., finite element solver) [119].

Equally important is the ability of SR to be bound on and validate physical laws [121]. For instance, Newton’s law of gravitation was somehow rediscovered [122], while another study has focused on rediscovering conservation laws [123], validated by a data-driven approach and given by a symbolic formula. However, care has to be taken when aiming on potential scientific discoveries, as oversimplified datasets and lack of evaluation metrics may lead to false results [124].

The investigation of complex and nonlinear dynamic systems demands deep understanding of the physical behavior in order to provide a reliable model [125]. Identifying differential equations via data-driven models, is a way to provide governing equations directly from data instead of physical laws, as equations in complex physical systems are scarce [126]. Nevertheless, issues may arise due to data scarcity or low fidelity, as oftentimes noise is present [80]. Noise can be, though, anticipated with Bayesian approaches. SR has also proven to be more effective than several ML models in small datasets [127] and by generating simple and physics-based relations, has an impact in its employability.

In contrast, the main drawback of SR is by no doubt the computational time needed for evaluating thousands or more equations [128]. This is one of the reasons that SR is better suited in applications where the number of input parameters is as small as possible [129]. To confront this barrier, a common strategy is to first identify the most important factors in the dataset (this can be done by other ML models, such as RF [101]) and then apply SR. However, this technique may affect the obtained results when accuracy is the main question.

From another point of view, as interpretability derives from the need to understand and trust an ML model, verify or manipulate it [130], and SR claims to be interpretable, at least compared to other black-box approaches, the degree of straightforwardness usually accounts for the size of generated expressions. Bloat is a common side effect that arises by GP, in which the results tend to suffer from a burst of complexity, while improvements on achieved fitting remain slight [131], though promising [131].

## Application in Science and Technology

Current computational techniques in most fields of science and engineering have been able to find a balance between computational efficiency and cost, as the rise of data-driven models, along with the introduction of parallel hardware architectures, constitute a novel framework that can be exploited to obtain accurate results faster. Taking further into account the inherent interpretability of SR models, the perspective of obtaining meaningful equations is welcomed by numerous fields in theoretical and applied science. An extended review on materials science applications will be made first, as it is an interdisciplinary field that enters most science and engineering fields, through material properties prediction and novel materials discovery, such as electrical and mechanical engineering, construction applications, biophysics and energy applications, to mention a few.

### Materials Science

Material science, from the sub-atomic to the macroscopic level, is currently undergoing a major shift towards full digitalization and automation and has opened new perspectives for innovation. Incorporation of databases, multi-scale computations, and experiments are integrated with the aim of reducing the time and cost of design and manufacturing of materials. AI techniques are now focused on finding new and/or predicting the properties of existing materials [25], which will make possible the discovery of novel, tailor-made materials. As materials investigation has been mainly conducted with expensive and complex experimental methods, under the particular researcher’s intuition [132], and theoretical analysis is widely based on empirical relations, by leveraging big data and AI methods, a new computational paradigm emerges, to pose as a catalyst for materials development, along with exploiting available simulation techniques. To this end, data from multiple-scales of microstructures can be embedded with physics-based descriptions, to reveal physical concepts such as thermodynamics, kinetics, functional and mechanical properties.

#### Multiscale Modelling

**Fig. 6:**
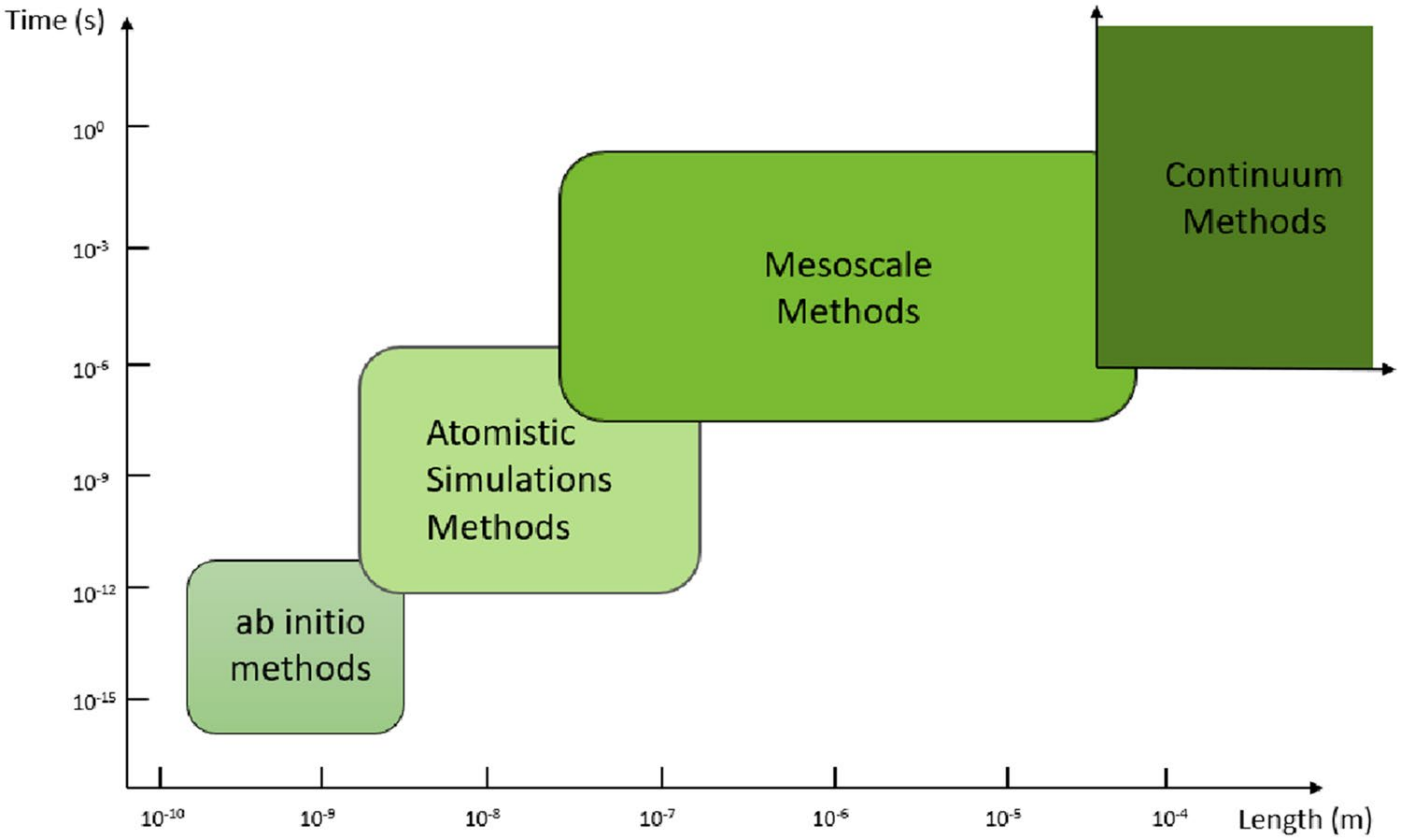
Multiscale modelling

Research on materials (solids or fluids) takes place at different scales of length and time, with each scale incorporating features from the former (see Fig 6).

Starting from the atomic scale, ab-initio methods (first principles) are performed by quantum mechanics (QM) calculations in order to obtain a form that describes the energy of a system, the potential energy surface (PES). Calculations are derived directly from physical laws and do not require the incorporation of any experimental data or assumptions. However, these are based on finding solutions to the Schrödinger’s equation, which may not be practical for most real-world systems. This can be partly anticipated by the incorporation of the density functional theory (DFT) [133]. Albeit capable of achieving quantum-accuracy, the usage of atomistic methods is limited in terms of the accessible computational time and simulation size. To overcome these barriers, a number of particle methods have arisen.

Particle methods include, among others, the methods of Molecular Dynamics (MD), Dissipative Particle Dynamics (DPD) and Smoothed Particle Hydrodynamics (SPH). These methods are appropriate for different size and time scales and share the same features with purely meshfree and Lagrangian nature. A particle in MD, DPD, and SPH acts as both a material point and as an approximation point, that is, the particle is regarded as a single atom or molecule in MD in atomistic scales, a small cluster of atoms or molecules in DPD in meso-scales, and a very small region in SPH in macro-scales [134].

By joining multiple techniques and methods, multiscale modeling has been developed and, during the past few years, it succeeded to integrate ML with simulations to create surrogate models [135, 136]. Research has shown that the construction of ML interatomic potentials (MLIPs) trained over ab-initio MD (AIMD) simulation results, could extend the possibilities of materials research by bridging DFT with MD and finite element (FE) simulations [137]. Furthermore, SR approaches thrive on interatomic potential by providing simple solutions that significantly decrease the risk of overfitting. Specifically, a SR model [138] generates fast and accurate many-body interatomic potentials formed by fundamental physical principles, which in turn are flexible enough to perform in multi-scale.

For example, a novel technique for bridging between micro- and macro-scale, Ref. [139], where a combination of computer simulations and ML models is suggested. Specifically, FE simulations were conducted for the generation of input data to enter the ML models, and, subsequently, ML algorithms develop a macroscopic model trained by a microscopic. The fact that traditional methods work in conjunction with ML models, provides the ability to bridge across scales and therefore provide more accurate predictions. However, it is also stated that analytical functions are appealing for the investigation of properties in micro- as well as in macro-scale [139]. Thus, SR appears as a prominent tool for multi scale investigation.

#### Properties Prediction

The development of material properties databases in a systematic way has altered materials research, as researchers opt for ML models to extract information out of them [26]. Both material properties and their relation to processing conditions, are translated to form new computational models [140]. There are cases where constitutive models express how a material responds in different conditions, which in turn produces a stress–strain relation to generate the governing laws [141]. For instance, data-driven plasticity formulas were generated in studies [142, 143], while in another approach [144], elastic solid models were constructed in similar fashion.

Moreover, several applications focus on equation generation from scratch towards the prediction of Lennard–Jones fluid diffusion [56, 145, 146] or the electrical conductivity of ionic liquids [101]. While others, have equipped SR to investigate lattice thermal conductivity [147], the critical temperature in superconductors [148] or the yield strength of polycrystalline metals by key physical quantities [149].

#### Discovery and Design

A concept familiar with material discovery, is material stability. There exist abundant element combinations one could perform towards information from the periodic table and produce a material, at least theoretically, since the additional criterion of stability, i.e., a measure to estimate if the hypothetical material is feasible, has to be taken into account. The role of SR in materials discovery and design has been evaluated [150] and it has been proven to be a suitable tool for the identification of compound representations (also known as descriptors [151]) and the creation of new that correlate with materials stability [152].

On the other hand, materials discovery and design, aim to fully exploit property prediction and produce materials with target behavior. Nevertheles, this might be a challenging task, as the targeted property could appear only in unique structures and, in addition, some properties ought to have a perfect alignment in order to achieve a high performance [153]. Therefore, it is vital to identify the parameters that govern the functionality and their dependencies, in order to optimize them [154].

A successful example of incorporating SR in such cases has been presented ref [152]. Without any prior knowledge on the problem’s domain, SR has generated accurate descriptors that define MXene stability. Furthermore, another study [155] has implemented SR and generated simple and meaningful descriptors that has ultimately contributed on the discovery of new catalysts. These applications illustrate the fact that SR can produce accurate descriptors without any chemical or other knowledge on the system and eventually accelerate the discovery of novel materials.

### Engineering Principles

In this section, various engineering sub-principles are to be evaluated on the applicability of SR-related methods, such as civil and construction, chemical, petroleum and natural gas engineering, mechanical and computer engineering. Main focus is the applicability of AI methods, and SR is capable to provide symbolic expressions to be used at hand and pose as a fast solution to real-life problems.

#### Construction and Building Materials

Starting from a subset of civil engineering, in hydraulics (liquid flows through pipes), the Colebrook equation for flow friction is a familiar model among engineers that is also being adopted by adjacent engineering disciplines [156]. Several models of the Colebrook equation via SR were generated in study [156], where the obtained results presented to be accurate enough. As the authors state, their approaches are only valid for a turbulent regime due to the fact that a transition from a laminar to a turbulent regime is not efficiently described by the Colebrook equation. They supplement, in their previous works, approaches were made by genetic algorithms and neural networks to model this transition unefficiently. In a subsequent study [157], simpler equations were discovered that unify laminar and turbulent hydraulic regimes and therefore diminishing the need to account for changes in flow patterns at separate laminar or turbulent flow models [157]. However, their dataset were generated by sampling through already established equations and not experimental data and therefore their application is hindered [157].

From another point of view, concrete, the main construction material for most higher-scale applications, has been also a popular subject of AI research. A number of published papers has been focused on the construction of models to estimate the seismic peak drift ratio [158, 159], the penetration depth into concrete blocks [160], the shear capacity of steel fiber-reinforced concrete beams, tracing fire response of concrete structures [161] or the seismic response through a fragility analysis [162], while others aim on the accurate description of remaining fatigue life [163, 164] or bearing-type bolted connections’ shear resistance. One should note that, while the investigation of previously noted instances were conducted by modelling measurements, in several occasions [160, 163, 165] the generated equations outperformed conventional employed formulas.

#### Chemical Engineering

Drag coefficient has a crucial role in gas-solid flows, as it provides an analytical view of the hydrodynamics therein [166]. At the industrial scale, Computational Fluid Dynamics (CFD) simulations are being employed towards this investigation [167]. Moreover, CFD simulations depend on the Euler–Euler or Euler–Lagrange models [167], which both of them need to possess efficient drag correlation models in order to take into consideration gas-particle interactions [168]. Current employment of SR techniques, are found in studies about the investigation of fundamental principles in the drag coefficient [169], construction of brand new drag correlations which can be used as input to CFD models [167] or a simple drag model [166] which have proven to outperform standard formulations.

Further notations highlight the prospect of SR employment to the catalysis field in order to obtain physic-based models [79], or its incorporation into a method to obtain a whey protein fouling prediction model in plate heat exchanger, by formulating a parameter that needs to be re-adjusted when a slight change on the solution of whey protein or process conditions take place [170]. Others, have successfully identified physical relations of fluids and kinetic laws of chemical reactions [171] or generated expression in order to predict the particle size distribution during fluidization [172].

#### Petroleum and Natural Gas Engineering

In petroleum engineering, oil viscosity is of significant value, with its calculation being conducted by experimental measurements or empirical formulas at different pressure regions [173]. However, the former occasionally suffer from inadequate measurement supply while the latter generates insufficiently outcomes [173]. To overwhelm this situation, in study [173], there is presented a SR approach in which correlation models were constructed across every pressure region directly from data points, which in turn managed to outperform current models. On the other hand, a SR application about predicting the rate of penetration in drilling of hydrocarbon reservoirs [174], resulted on an expression to be overpowered by RF and ANN estimations [174].

Moreover, foam induced by a surfactant solution and nitrogen, finds room of application in tasks such as oil recovery, acid diversion and aquifer remediation, with its mobility being generally characterized in terms of pressure drop [175]. Capturing the physical behavior of the system and classifying relative variables according to their significance to steady state pressure drop, was accomplished by generating analytical expressions in study [175], by accepting no prior knowledge regarding the underlying physical behavior. Others, have focused on modelling oil production [176] or estimating the multiple fractured horizontal wells flow performance [177].

In contrast to other hydrocarbon-based materials (e.g., oil, coal), natural gas constitutes a cheaper and cleaner option [178] to meet our energy demands. Similar to petroleum engineering, estimating the viscosity is one of the top priorities in natural gas studies, as it can be utilized to efficiently synthesize models about production, transportation or gas storage systems [179]. To this end, SR studies regarding the prediction of dynamic viscosity [180] or pure and impure viscosity [179] appear most appealing. Supplementary applications, deal with models construction towards hydrate formation temperature estimation [181], estimation of equilibrium water dewpoint temperature [182] and the prediction of the gas compressibility factor [178].

#### Mechanical Engineering

Objectives of control systems could be summarized into maintaining a process, which could be affected by external parameters, and a transition from one process to another [183]. In order to do so, the control system often manages other parameters (e.g., pressure, temperature, etc.) to reach or preserve a certain status [183]. In this regard, several studies have equipped SR to generate analytic functions towards a control system design [128, 184].

Moreover, SR has been utilized to derive models capable of describing the underlying connection between alloy composition, cooling time and hardness, in welding heat-affected zone of low alloy steel [185]. Additionally, SR has been employed to estimate constitutive model parameters in an alloy research [186], or even facilitated to create expressions to search for the optimized components during machine tool design by finding the modal mass distribution matrix, which is usually hard to obtain [187].

Furthermore, SR has provided constitutive formulas of material behavior in aluminum alloys [119] or been incorporated into a mergent of techniques where SR estimated the calibration parameters of a physics-based model [188]. Calibration of model parameters that depend on processing conditions may pose a major obstacle [140]. These parameters are occasionally fitted, in order to reach an agreement with measurements; for that reason, the degree of importance of other parameters on the calibration parameters is not completely established [188]. To overcome this issue, two techniques have been proposed, the explicit and implicit [140].

In the explicit method, the calibration parameters are primarily optimized, while a formula for the prediction of the optimized values using SR, is generated next. Then, a combination of the generated expressions on calibration parameters and a physics-based constitutive model takes place, in order to create a hybrid approach. In the implicit method, no optimization of calibration parameters occurs as they undergo a tree-based GP procedure on the first steps. In addition, no extra combination of expressions similar to the explicit method are performed, as they are already combined in a multi-tree GP, where each individual has a number of trees that correspond to the number of calibration parameters. Note, that the authors recommend the implicit method for further use, while they also note that although the implicit method may be more computationally expensive, the remarkably higher accuracy cannot be ignored.

#### Computer Engineering

Computer and Information science has much to offer to SR programming, from the view of constructing and suggesting new techniques to improve the applicability of the genetic algorithms on existing physical and industrial problems. For example, a novel fault detection mechanism has been constructed with SR symbolic techniques and found to achieve better results than traditional methods, such as the support vector machine and pattern recognition neural network algorithms [189]. Moreover, another industrial application refers to enhancing models of learning behavior that present, better learning response than manual and experienced learners [190]. Techniques such as the narrowing of the search space through a semantic cluster library have given promising results [191], while a statistical-based SR algorithm has been proposed that uses statistical information to improve its performance [192].

On the other hand, SR improvement may be also achieved by decomposing the problem under investigation into several subproblems [193]. There are also cases where SR has been bound to reinforcement learning, and has been able to deal with dynamic tasks, with back-propagation capability [194] or even a dynamic process formulation [195]. Finally, since GP problems oftentimes require tons of computational time to complete, the evaluation time has been used as an estimate of model complexity and a new method is proposed to control it [196].

### Other Fields

#### Physics and Astronomy

Data from astronomical observations is undoubtedly rich and AI methods are well-posed to its exploitation. For example, galaxy clusters turn out to be the most immense structures in the universe [197], as they contain several galaxies, that further include dark matter, black holes and more [198]. Moreover, they operate by mechanisms regarding the evolution and formation of those, whose details are not yet fully understood [198]. Thus, various approaches have emerged for their investigation, such as searching of expressions capable to unify properties of galaxy clusters to their masses [197], studying the galaxy-halo connection [199], modelling the assembly bias [129] and estimating the total mass of a subhalo [198].

In addition, SR has been applied in exoplanet transit spectroscopy modelling [200], as observations of planetary transits at different wavelengths are often investigated as a method to gain knowledge of the structure and composition of an exoplanet’s atmosphere [200]. Further applications worth noting, include rediscovering orbital anomalies from observations of position and velocity by generating a model of dynamics [201], predicting gravitational waveform surrogates [202], reconstructing the duality parameter in an approach to predict the cosmic distance duality relation with strongly lensed gravitational wave events [203], analyzing solar activity in a solar cycle and successfully revealing underlying governing laws regarding magnetic wave generation [204].

#### Energy and the Environment

Green growth is the way towards a sustainable future. Although SR cannot be considered as the basic means of directing the world towards green transition, it can be easily implemented to contribute to a greener environment. For example, an SR-based study supports that green transition is highly likely to be embraced by developed countries, while underdeveloped or still developing countries follow a model where they choose economic growth over green [205]. The current work avoids classification of countries in developed or underdeveloped and focuses on ways to enhance the green environment’s harnessing systems and fundamental components that it is accounted for.

Energy from the sun is currently covering a substantial amount of global energy demands. From a ML point of view, applications such as solar radiation prediction [206, 207] and photovoltaic power prediction [208] can open the pathway towards better energy management. Apart from renewable applications that incorporate solar radiation, wind energy is also harnessed to produce a significant amount of energy resources. Wind speed analysis [209, 210] and wind power generation efficiency [211] studies, could eventually produce an improved version of wind turbines, by exploiting novel computational ML models.

However, from an environmentalist point of view, an energy surplus should not lead on an excessive use of available energy, by continuously increasing the income of generated power to adhere to our costly way of life; damage should be also minimized. Damage minimization can occur via energy consumption modelling [90, 212, 213], energy management [214], carbon emission studies [215, 216], exhaust emission [217] and modelling of air quality [91]. All these applications can contribute towards a green energy transition and securing a healthier planet.

#### Medical Sciences

The majority of SR applications in medical science, follow a similar pattern, starting from the identification of important features between a dataset (usually measurements or patients’ medical history) and followed by the establishment of suitable models towards forecasting, prognostication or successful diagnosis.

For example, in a study about Parkinson disease [218], SR was able to find important features that relate to gait changes. Similarly, in study [219], a comprehensible risk model was generated to predict survival rate of breast cancer patients. Further applications are found in analyzing measurements towards hepatocellular carcinoma diagnosis (liver cancer) [220], estimating hemoglobin and glucose levels in blood by modelling key features from fingertip videos [221], pairing patients that show similar characteristics on the way to radiotherapy dose reconstruction and therefore improve the design of radiation treatments [222] or analyzing measurements of different body areas towards human walking modelling [223].

Additionally, SR incorporation has led to the enhancement of previous estimator models, adding mathematical interpretability to previously adopted “black-box” ML models [224]. More specifically, SR has been exploited to create mathematical formulas about transformations of covariates from patients’ medical records and then those formulas were used in the Cox model [225], resulting in even higher prediction accuracy compared to solely applying the Cox model. In another study about pregnancies which develop pre-eclampsia, SR has outperformed models based on logistic regression by identifying relations between important features [226].

#### Financial

The Covid-19 pandemic wreaked havoc to previous macroeconomic models, and thereupon the need to establish accurate estimates is now, more than ever, evident [227]. Macroeconomic models are often utilized as a means to guide political and financial decisions [228] and by integrating SR into those approaches, possible relations between variables might come up to light. Early studies have focused on the recognition of those interactions in large datasets that contain different observations of several economic quantities [228], while succeeding models are centered in the prediction of crude oil price [229] and economic growth forecasting by investigating expectations of agents. Agents’ expectations regarding the economy’s condition are high-valued for economic modelling due to the fact that they contain explicit, multi-variate information about market [230] and usually obtained via tendency surveys (business and consumer surveys) [231]. As a result, approaches are made via SR to form a link between survey data and a successful economic growth model [227, 232].

Another application of SR via GP has been in econometric modelling [233] where the conventional ”exchange equation” was reinvented. However, GP has not revealed its full prospective in the field, as being underused in macroeconomic modelling [227]. Researchers think that the polymeric generated expressions, who differ by those European Commission presents [232], will eventually escalate the situation by proving their superiority and update the currently equipped models.

## Conclusion and Future Perspectives

Symbolic Regression has emerged as a method that bridges the gap between data, ML models and scientific theories, providing analytical equations at hand, and this can be specifically applicable in cases where only empirical and numerical approaches have been established. Barriers that may appear lie in the increased computational cost, equation complexity, and efficient programming approaches.

It is imperative that new computational tools be exploited in the future in order to reduce the computational cost of SR. As advanced NN architectures and functions are constantly being proposed for complex DL tasks, they should also be embedded in an SR framework. For example, the incorporation of Generative Adversarial Networks along with the SR algorithm, would facilitate the discovery of hidden physics in small datasets. Data pre-processing is also an open issue, since there is no standard procedure to deal with raw data, as is the case with common ML tasks. Moreover, parallel, GPU-based implementations would accelerate the SR process, reducing computational time, which, in cases of many-input datasets, is prohibitive.

The transformational impact of SR on science and technology can be very high. Notwithstanding the fact that results coming from SR investigation in various fields, both in research and applied sciences, are still preliminary, the method has shown great potential, extracting models with performance comparable to state-of-the-art empirical relations widely used in the literature.

Novel algorithms, efficient data preprocessing methods, data mining, and new function prediction, bound to physical laws and as simple as possible, should be further prospected to guide future scientific research. Following disciplines that adhere to generalizability, interpretability, and applicability, SR is the AI branch that will be on the focus of primary research in the years to come and its evolution will be towards meeting the requirements of practical applications.

We should keep in mind, however, that SR is not directed to replace well-established theoretical research and mathematical approaches. It is more likely to be bound on data science techniques and suggest an alternative method of explaining and discovering hidden patterns and behaviors in data coming from various sources. The concept of obtaining an analytical expression to describe physical phenomena and processes only by diving into a dataset is practical and can be intriguing, but, on the other hand, we believe that many things are yet to be done on the direction of ensuring that this expression is physically explainable and lie on firm basis.

## Supplementary Information

Below is the link to the electronic supplementary material.

Supplementary material 1 (PNG 27.0 kb)

Supplementary material 2 (PNG 30.4 kb)

Supplementary material 3 (PNG 20.7 kb)

Supplementary material 4 (PNG 44.6 kb)
