# Supplementary figures and images for: Artificial Intelligence in Physical Sciences: Symbolic Regression Trends and Perspectives
Source: Arch Comput Methods Eng. 2023 Apr 19:1–21. Online ahead of print. doi: 10.1007/s11831-023-09922-z (PMC10113133; doi:10.1007/s11831-023-09922-z)

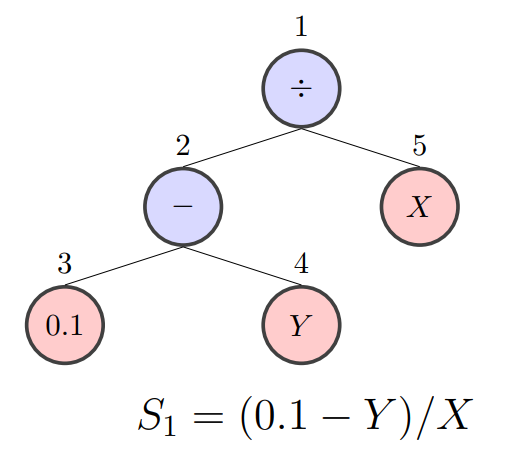

Supplement: Supplementary file 1 — Supplementary material 1 (PNG 27.0 kb) [file 11831_2023_9922_MOESM1_ESM.png]

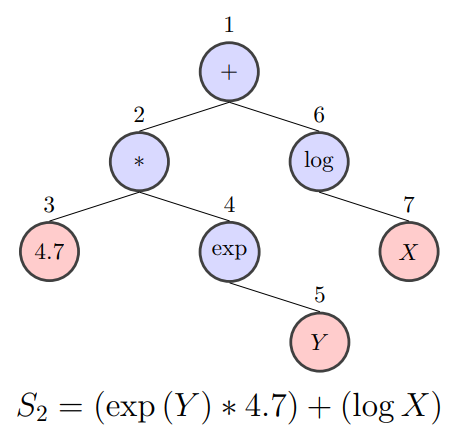

Supplement: Supplementary file 2 — Supplementary material 2 (PNG 30.4 kb) [file 11831_2023_9922_MOESM2_ESM.png]

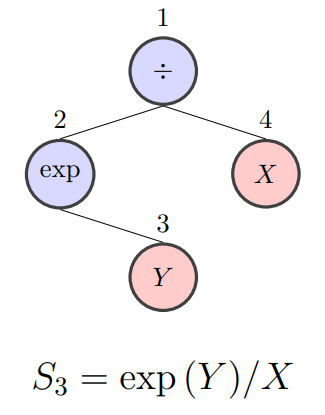

Supplement: Supplementary file 3 — Supplementary material 3 (PNG 20.7 kb) [file 11831_2023_9922_MOESM3_ESM.png]

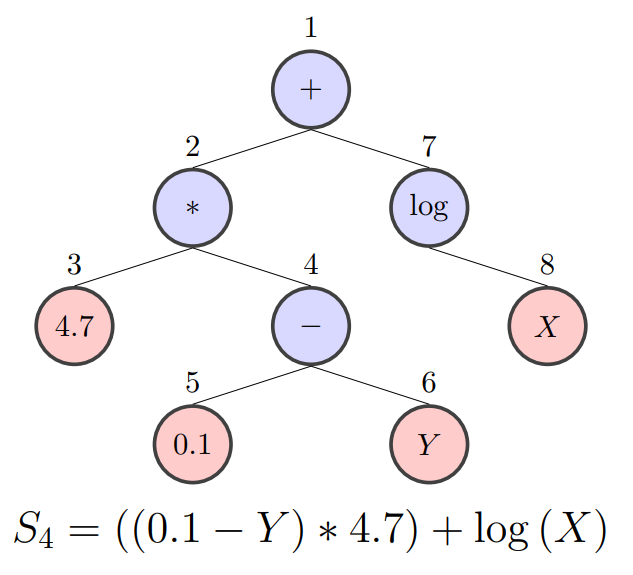

Supplement: Supplementary file 4 — Supplementary material 4 (PNG 44.6 kb) [file 11831_2023_9922_MOESM4_ESM.png]
